# Gastric trichobezoar

**DOI:** 10.1002/ccr3.1213

**Published:** 2017-09-29

**Authors:** Daniel José Szor, André Roncon Dias

**Affiliations:** ^1^ Hospital das Clínicas University of São Paulo São Paulo Brazil

**Keywords:** Laparoscopy, minimally invasive surgery, trichobezoar, trichophagia

## Abstract

Trichobezoar is a rare gastrointestinal pathology, but should be considered in patients with abdominal mass and previous history of trichophagia. In physical examination, it is important to verify signs of alopecia. Minimally invasive surgery is a secure method to remove the specimen from the stomach.

Question: What does this surgical specimen represent?

Answer: This is a gastric trichobezoar surgically removed from a 14‐year‐old‐patient with previous history of trichophagia. The patient presented in the emergency room with a previous story of epigastric pain and abdominal distension for the last 6 weeks. Abdominal computed tomography was performed and suggested trichobezoar, confirmed by upper endoscopy, which was unable to remove the specimen. As the patient was symptomatic, surgical intervention was indicated. A laparoscopic gastrostomy was performed, and the specimen was removed from the abdominal cavity toward a Pfannenstiel incision (Fig. 1). The patient was discharged 3 days after surgery without complications and present follow‐up is 8 months.

**Figure 1:**
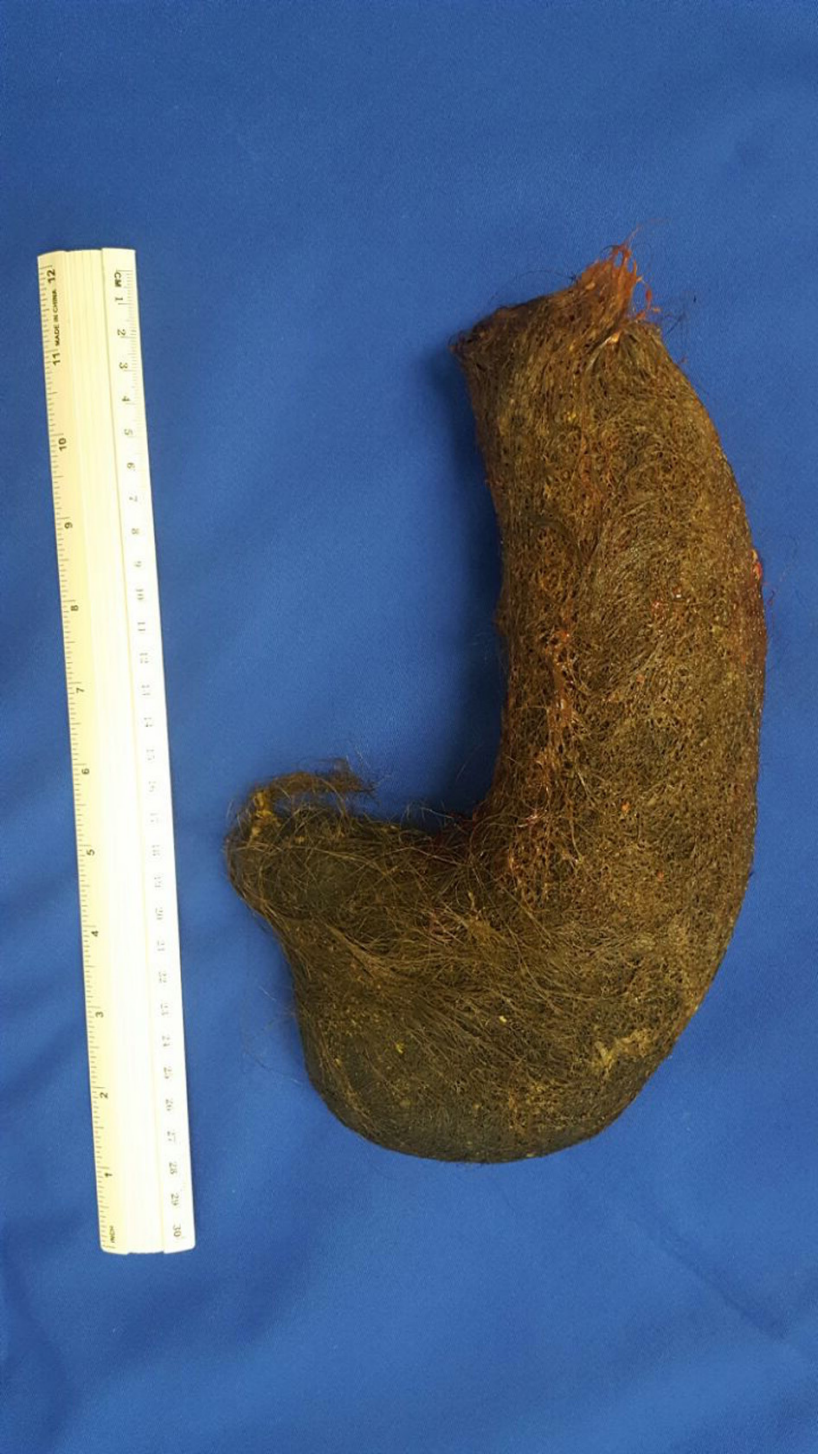
Gastric trichobezoar.

## Authorship

DJS: prepared the manuscript, member of surgical team. ARD: reviewed the article, member of surgical team.

## Conflicts of Interest

None declared.

